# Investigation the Cytotoxicity of 5-AZA on Acute Lymphoblastic Leukemia Cell Line In Vitro and Characterization the Underlying Molecular Mechanisms of Cell Death and Motility

**DOI:** 10.31557/APJCP.2021.22.11.3723

**Published:** 2021-11

**Authors:** Maryam Moghadasi, Ali Mohammadi, Behnam Emamgolizadeh, Mohammad Reza Alivand, Dara Rahmanpour, Saeed Solali

**Affiliations:** 1 *Student Research Committee, Tabriz University of Medical Sciences, Tabriz, Iran. *; 2 *Department of Immunology, Division of Hematology, Faculty of Medicine, Tabriz University of Medical Sciences, Tabriz, Iran. *; 3 *Department of Cancer and Inflammation Research, Institute of Molecular Medicine, University of Southern Denmark, Odense, Denmark. *; 4 *Department of Medical Genetic, Tabriz University of Medical Sciences, Tabriz, Iran. *; 5 *Molecular Medicine Research Center, Tabriz University of Medical Sciences, Tabriz, Iran. *

**Keywords:** 5-AZA, acute lymphoblastic leukemia (ALL), DNA methylation, microRNA, apoptosis

## Abstract

DNA methylation is a reversible biochemical process determinant of gene expression that is frequently observed in acute lymphoblastic leukemia (ALL). This is believed to arise from aberrant DNA methyltransferase activity establishing abnormal levels of DNA methylation in tumor cells. DNA methyltransferase inhibitor, 5-azacytidine (5-AZA), is a clinically used epigenetic drug which induces promoter demethylation and gene re-expression in human cancers. In this study, we investigated the cytotoxicity of on MOLT4 and Jurkat leukemic cell line in vitro and characterized the underlying molecular mechanisms of cell death and motility. MOLT4 and Jurkat cells were treated with 5-AZA for 12, 24 and 48 hours. The effect of the 5-AZA treatment on cell viability (MTT assay), apoptosis (annexin V/PI staining), microRNA (miRNA) and mRNA expression (real-time PCR) was measured. The results showed that 5-AZA could induce MOLT4 and Jurkat apoptotic cell death in vitro in a time-dependent manner and probably via apoptotic mechanisms. We found that treatment with 5-AZA could increase the expression of epigenetically silenced miRNAs, miR-34a, miR-34b and miR-124-1 in treated cells. In addition, mRNA analyses demonstrated that MOLT4 and jurkat cells, expressed p53 gene more than 10-fold higher compared with untreated cells in three independent experiments while the cells suppressed the expression of a subset of functionally related genes including MYC, BCL2, APEX, SIRT1, SNAIL1 and vimentin to some extent, following 5-AZA treatment. We found that a miRNAs expression level in treated cell lines was closely correlated to the expression of their target genes. Together, these findings suggest that 5-AZA may affect the viability of MOLT4 and jurkat cells, at least in part, by regulating the transcription of genes that are associated with cellular apoptotic response.

## Introduction

Acute lymphoblastic leukemia (ALL) is the most common malignancy diagnosed in children and the second most common leukemia of adults. It accounts for 75% of all acute leukemias in children where the overall cure rates of 80% are achieved. However, for adults with ALL, pediatric protocols of treatment are insufficient to achieve disease remission with only 30-40% long-term survival. Chemotherapy is considered to be the main approach in the treatment of ALL. Current chemotherapy for ALL patients consists of induction (first), consolidation and maintenance phases of treatment (Ball and Stein, 2019). The role of induction chemotherapy has been to reach a remission and thereby improving curability. After induction therapy, transplant-eligible patients are offered allo-SCT while the majority of patients go on to consolidation and maintenance chemotherapy (Terwilliger and Abdul-Hay, 2017). Consolidation chemotherapy may help to eradicate the remaining leukemia cells and hence decrease the relapse rate after the induction regimen. Allogeneic stem-cell transplantation (allo-SCT) is currently the standard of care for patients with ALL who progress on first-line chemotherapy (Mactier and Islam, 2012), but it can still cause substantial mortality and morbidity (Crotta et al., 2018). It has long been assumed that response to initial therapy predicts additional therapy outcome. Especially, ALL patients who relapse ‘early’ after initial chemotherapy fail to response very effectively to allo-SCT (Crotta et al., 2018). In addition, results of intensive chemotherapy of ALL patients also appears disappointing (Dombret and Gardin, 2016). Therefore, further research is needed to develop effective new agents to be integrated into the treatment plan of patients with ALL.

Epigenetic modifications, particularly DNA hypermethylation have been established as a key event in the development of ALL that result in transcriptional gene silencing. In addition, DNA methylation status is one contributory factor in the treatment of ALL cancer (Burke and Bhatla, 2014; Nordlund and Syvanen, 2018). The altered DNA methylation as a hallmark of many cancer types is believed to be associated with the deregulation of DNA methyltransferases (Rahmani et al., 2017). 5-AZA is a chemotherapy drug analogue of the nucleoside cytidine which is thought to induce antineoplastic activity via binding with DNA methyltransferase 1 (DNMT-1) causing hypomethylation of DNA and direct cytotoxicity when incorporated into RNA and DNA (Gravina et al., 2010; Khan et al., 2012). The hypomethylating agents (or DNA methyltransferase inhibitors), 5-AZA and decitabine, were first studied in higher-risk myelodysplastic syndrome (MDS) and acute myeloid leukemia (AML) as front-line therapy for patients in whom allo-SCT is not a feasible option. The advent of demethylating agents has greatly advanced the treatment of myeloid malignancies (Platzbecker et al., 2012). Treatment with 5-AZA may also be preferred over intensive chemotherapy in these cases. 

Studies in vitro and in vivo have shown that 5-AZA is able to restore the expression of epigenetically-silenced tumor suppressor genes (Gailhousteet al., 2018). Tumor-specific downregulation of several microRNA (miRNA) species has been observed in virtually every type of human neoplasia and contributes to the establishment of these malignancies. Aberrant promoter methylation of tumor suppressor *miRNA* genes has been detected as an early driver event in ALL leukemogenesis. miRNAs are small regulatory molecules involved in multiple cellular processes, including proliferation, cell cycle regulation and apoptosis (Adams et al., 2014). Methylation in ALL cells is implicated in the inactivation of the genes playing a role in the control of three key cellular pathways: growth deregulating events at late G1 cell-cycle checkpoint and G2-M transition, the apoptotic program and the cell-cell adhesion. The two putative tumor suppressor miRNAs, miR34a and miR34b have been shown to induce cell cycle arrest and apoptosis (Hermeking, 2010). It is demonstrated that three members of the miR-34 family, including miR-34a, b and c, are all direct targets of p53 protein. Recent reports point that miR34a and miR34b are often hypermethylated and hence silenced in the majority of patients with chronic lymphocytic leukaemia (CLL) (Wang et al., 2014; Wong et al., 2011). These two tightly linked miRNAs are associated with a critical network of genes that are related to the control of cellular proliferation (MYC, cyclins and cyclin-dependent kinases), apoptosis (BCL2, SIRT1) or motility (SNAIL1, vimentin) all of which are linked to tumorigenesis and cancer progression (Ito et al., 2017; Misso et al., 2014; Rokavec et al., 2014).

miR-124 is one of most commonly deregulated miRNAs in cancers of the breast, lung, brain, etc. There are three miR-124 genes (*miR-124-1, miR-124-2, *and *miR-124-3*) in human genome. miR-124-1 is a highly conserved miRNA which is hypermethylated in myeloma and lymphoma cell lines but not in normal blood cells indicating that the miR-124-1 methylation is a tumor-specific phenomenon (Wong et al., 2011). Increasing studies have shown evidence of reduced miR-124-1 expression in the course of tumor initiation and progression and it is recognized to act as a tumor suppressor in haematological malignancies (Chen et al., 2014; Wong et al., 2011). Studies have shown that overexpression of miR-124 significantly suppresses tumor growth and invasion, and promotes apoptosis of the cancer cells in many human malignancies, but little is known concerning the role of miR-124 in ALL.

A number of compounds that target these tumor-associated miRNAs are already in preclinical development (Azimi et al., 2016). However, the effect of 5-AZA on these miRNA expressions and the downstream pathways in ALL is not yet known. The aim of this work was to explore the potential antiapoptotic effects of 5-AZA and further the modulation of growth-related miRNAs and tumor suppressive mechanisms in the MOLT4 and jurkat cell lines.

## Materials and Methods

*Cell culture and treatment with 5-AZA*

The adult T-ALL MOLT4 and Jurkat cells were obtained from the Pasteur Institute, Tehran, Iran and cultured in RPMI 1640 medium (Gibco) supplemented with 10% fetal bovine serum (FBS) (Gibco), and penicillin-streptomycin (100 U/ml penicillin and 100 μg/ml streptomycin). Cells were grown at 37°C in a 5% CO_2_ humidified atmosphere and treated with 1, 5, 10, 20, 30 and 40 mM of 5-AZA 24 hours after plating. The stock solution of 5-AZA was prepared in dimethyl sulfoxide (DMSO) at a concentration of 100 mg/ml and further diluted to yield the indicated concentrations. The assays were conducted pre-treatment and at 12, 24 and 48 h after the 5-AZA treatment.

*Cell viability assay*

MOLT4 and Jurkat cells were seeded in 96-well plates with a density of 20 000 cells per well. Cell viability was assessed 12, 24 and 48 h after the initial drug treatment using a colorimetric assay with 3-(4,5-dimethyl-2-thiazolyl)-2,5-diphenyl-2H-tetrazolium bromide (MTT). The MTT assay is an indicator of mitochondrial respiratory chain activity of viable cells based on the reduction of soluble yellow MTT tetrazolium salt to a blue insoluble MTT. After the treatment period, MTT test was performed according to the method previously reported (Naimi et al., 2019). The cell survival was expressed as the ratio of absorbance in the sample well to that of the control. The IC_50_ values typically defined as the concentration of 5-AZA required for 50% inhibition of the cell growth were obtained graphically by GraphPad Prism version 7.04 for Windows (GraphPad Software, La Jolla CA; www.graphpad.com). All experiments were conducted in triplicate and repeated three times. 

*NA extraction and real-time PCR*

Total cellular RNA from cultured MOLT4 and jurkat cells was isolated 12, 24 and 48 h post treatment with 5-AZA by use of TRIZOL reagent (RiboEx,Gene All) and the acid guanidinium thiocyanate-phenol-chloroform (AGPC) extraction method (AGPC) following the manufacturer’s protocol. At each time-point, total RNA was extracted to evaluate the relative expression levels of miRNA and candidate target genes by real-time polymerase chain reaction (PCR). For this purpose, complementary DNA (cDNA) was synthesized from RNA using PrimeScript™ reagent Kit (RevertAid™, First Strand cDNA Synthesis Kit). For miRNA analysis, cDNA was obtained using miRNA cDNA synthesis kit (Invitrogen, USA) from DNA-free total RNA as described by Azimi et al., (2016). For subsequent gene expression analyses quantitative real-time PCR was performed in duplicate for each sample using the RealQ Plus 2x Master Mix Green (Ampliqon, Danmark). Specific primer sequences for *BCL2, MYC, APEX, p53, SIRT1, SNAIL1*, vimentin and the corresponding miRNAs are shown in Table1. GAPDH was employed as internal control gene.

*Flow cytometry for detection of apoptosis*

The impact of 5-AZA on cell apoptosis was examined by flow cytometry with the Annexin V/PI (propidium iodide) assay according to the protocol of the Apoptotest™-FITC Kit (EXBIO, Slovakia) at different time points. The Annexin V/PI assay appears to determine which cell death pathway is involved in the cytotoxic effects in cultured MOLT4 and Jurkat cells. In this assay, annexin V has the biological property of binding to phosphatidylserine-containing membranes and PI is a DNA-binding fluorochrome which penetrates damaged cellular membranes and therefore, the assay is considered to discriminate between early apoptotic cells and late apoptotic or necrotic cells (Šatkauskas, Jakštys, Ruzgys, and Jakutavičiūtė, 2016). For each sample, 1 × 10^5^ cells were stained with 5 µL AnnexinV-FITC and 5 µL of PI and incubated for 15 min at room temperature in the dark, then immediately analyzed by the FACSCalibur flow cytometer (BD Biosciences). All tests were performed in triplicate and analyzed using FlowJo software Version 10.4.1 (Treestar, FlowJo).

*Statistical analysis*

Student’s t-test was used for a comparison between each pairing group. All numerical data are presented as means ± SEM of three independent experiments. P values < 0.05 were considered to be statistically significant.

## Results

*5-AZA has cytotoxic activity against MOLT4 and Jurkat cells*

**Figure 1:**
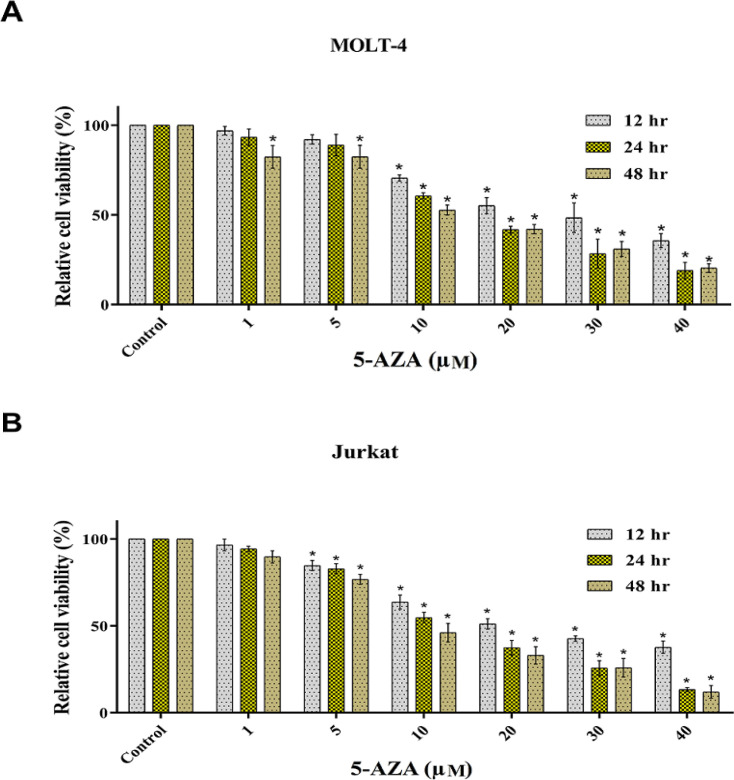
MOLT4 (A) and Jurkat (B) Cells Viability Assay. The MOLT4 and Jurkat cell viability was determined in all experimental groups using the MTT assay upon 12, 24 and 48 hours of treatment with 1, 5, 10, 20, 30, and 40 µM 5-AZA. Student’s t-test was used for a comparison between each pairing group. All numerical data are presented as means ± SEM of three independent experiments. P values < 0.05 were considered to be statistically significant. 5-AZA; 5-azacytidine; SEM, standard error of the mean

**Figure 2:**
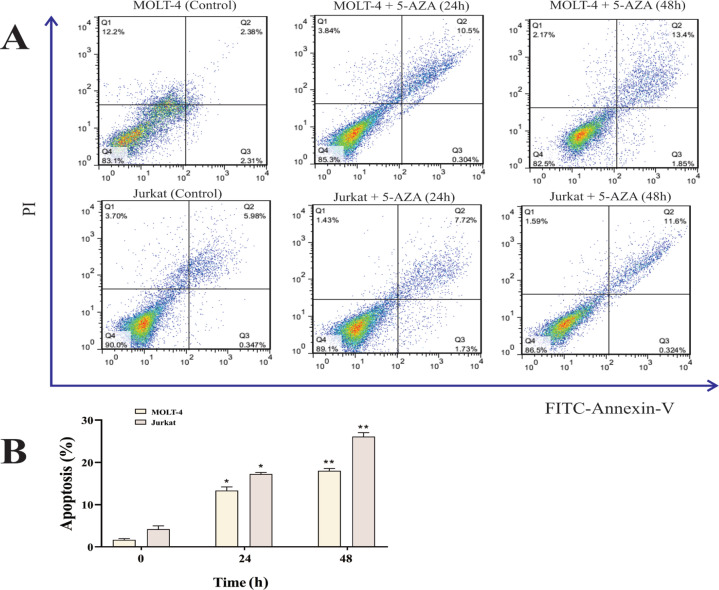
Measurement of apoptotic cells in MOLT4 and Jurkat cells at 24 and 48h of incubation. Figure 2A demonstrates a representative sample of assessment of apoptosis using Annexing-V/PI staining following cells exposure with IC50 concentration of 5-AZA. The MOLT4 cells were treated with 16.51 and 13.45 µM of 5-AZA at 24 and 48 hours of treatment , respectively. The Jurkat cells were treated with 12.81 and 9.78 µM of 5-AZA at 24 and 48 hours of treatment, respectively. The data presents the three independent experiments. Percentage of cells in each quadrant is shown (viable cells are in Q4 and apoptotic cells are in Q2 andQ3). Figure 2B shows apoptotic cells percentage induced with IC50 concentration of 5-AZA. The graph presented as figure 2B represents the average of the 3 experiments of which a representative one is shown in 2A. Data is presented as means ±SEM of three independent experiments. Student’s t-test was used to assess the observed statistical differences. P values <0.05 were considered statistically significant. (* ; p < 0.05, ** ; p < 0.01), 5-AZA; 5-azacytidine; SEM, standard error of the mean

As shown in Figure 1A, we treated MOLT4 and Jurkat cells with 5-AZA at six different doses, and the number of viable cells was quantitated by the MTT assay and compared to control cultures containing no drug within 12, 24 and 48 hr of incubation. A dose dependent inhibition was noticed for cell growth in the presence of 5-AZA (Figure 1A, B). The IC_50_ value of 5-AZA ranged from 10 to 20 µM and the median IC_50_ value at the 24 and 48 hr of incubation in MOLT4 cells were 16.51 and 13.45 µM and in jurkat cells were 12.81 and 9.78 µM, respectively. Analysis by flow cytometry using PI/annexin V-FITC staining was employed to determine the frequency of apoptotic cells. At 24 hours of treatment of MOLT4 cells with 16.51 µM of 5-AZA and at 48 hours of treatment of MOLT4 cells with 13.45 µM of 5-AZA, we found significant increase of apoptosis compared to controls (Figure 2A, B). Apoptosis percentages in MOLT4 cells (control), cells at 24 hours of treatment and cells at 48 hours of treatment were 2.11±1.13, 13.93±2.85, 18.29±2.18 % of total cells, respectively (Figure 2A, B). Moreover, there was noticeable promotion of apoptosis compared to controls at 24 hours of treatment of Jurkat cells with 12.81 µM of 5-AZA and at 48 hours of treatment with 9.78 µM of 5-AZA (Figure 2A, B). Apoptosis percentages in Jurkat cells (control), cells at 24 hours of treatment and cells at 48 hours of treatment were 4.31±2.10, 17.91±6.85, 28.11±2.44 % of total cells, respectively (Figure 2A, B).

*5-AZA treatment induces reexpression of the silenced miRNA genes in treated cells*

**Figure 3:**
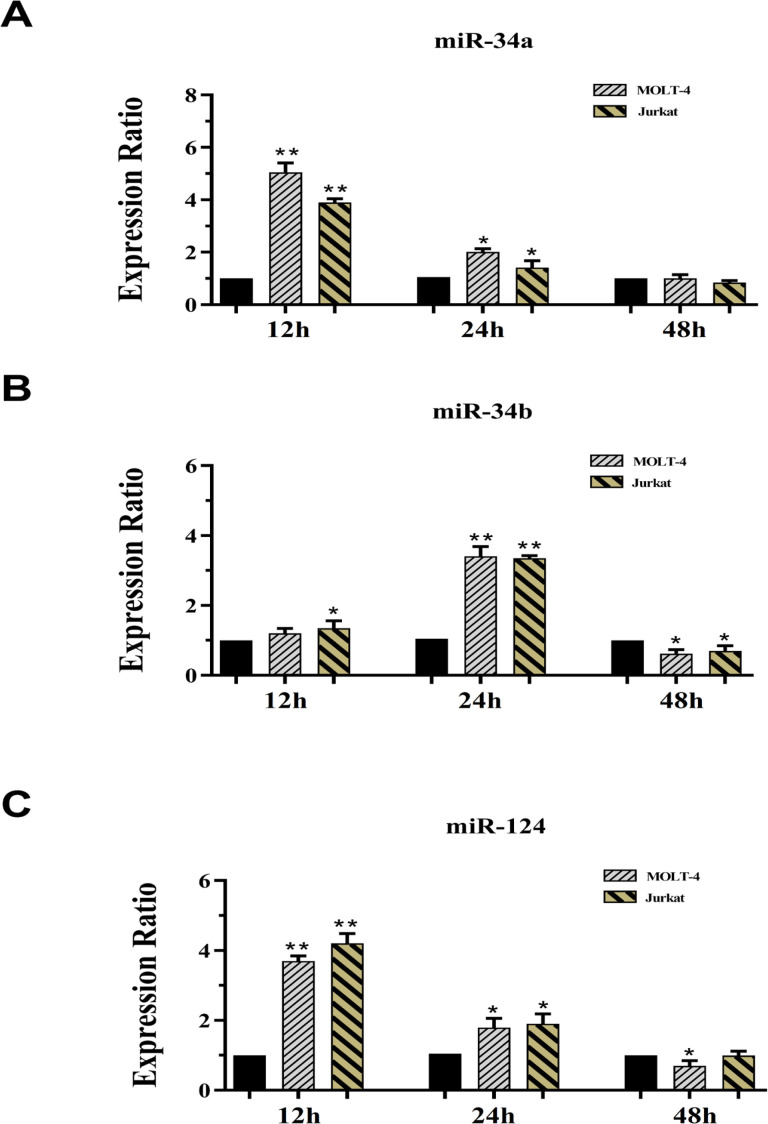
Real Time-PCR Data for miR-34a (A), miR-34b (B) and miR-124-1 (C) Expression. Expression levels of the candidate’s miRs were evaluated in MOLT4 cell after exposure with 16.51µM 5-AZA and in Jurkat cell after treatment with 12.81µM 5-AZA at 12, 24 and 48 hours of treatment. GAPDH was selected as internal control. Student’s t-test was used for a comparison between each pairing group. All numerical data are presented as means ± SEM of three independent experiments. P values < 0.05 were considered to be statistically significant. (* ; p < 0.05, ** ; p < 0.01), 5-AZA; 5-azacytidine, SEM; standard error of the mean

In this study, we have examined a set of epigenetically regulated miRNAs which target a multitude of genes involved in cell cycle regulation, apoptosis and invasion. Recently, dysregulation of the* miR-34* family of *miRNA *genes by DNA methylation is suggested to be a master regulator of tumor suppression that is associated to p53-dependent apoptosis. The miR-124-1, a highly methylated tumor suppressor, generally inhibits cell proliferation and can also repress multiple pro-metastatic targets (Lv et al., 2011; Shi et al., 2013). This aberrant methylation may be reversed by hypomethylating treatment, restoring miRNA-expression levels and rescuing the tumor phenotype. In order to evaluation of 5-AZA effect on target miR expression, MOLT-4 cells were treated with 16.51 µM of 5-AZA and Jurkat cells were treated with 12.81 µM of 5-AZA within 12, 24 and 48 h of exposure (Figure 3). According results, 5-AZA was able to promote expression levels of miR-34a in MOLT4 and Jurkat cells within 12 and 24 h, but not 48 h of treatment (Figure 3A). This enhancement in 12 h was more prominent than 24 h of incubation (Figure 3A). Also, expression level of miR-34b in Jurkat cells was improved within 12 and 24 h of incubation and attenuated within 48 h of treatment compared with control cells. Although expression level of miR-34b in MOLT4 cells was enhanced within 24 h of incubation and declined within 48 h of treatment compared with control cells, it was not modified at 12 h of treatment (Figure 3B). While 5-AZA had the potential to increase expression levels of miR-124-1 in MOLT4 and Jurkat cells within 12 and 24 h of incubation, expression level of miR-124-1 was declined in the MOLT4, but not Jurkat cells within 48 h of incubation (Figure 3C).

*5-AZA treatment can modify the expression of genes regulating apoptosis in treated cells*

**Figure 4:**
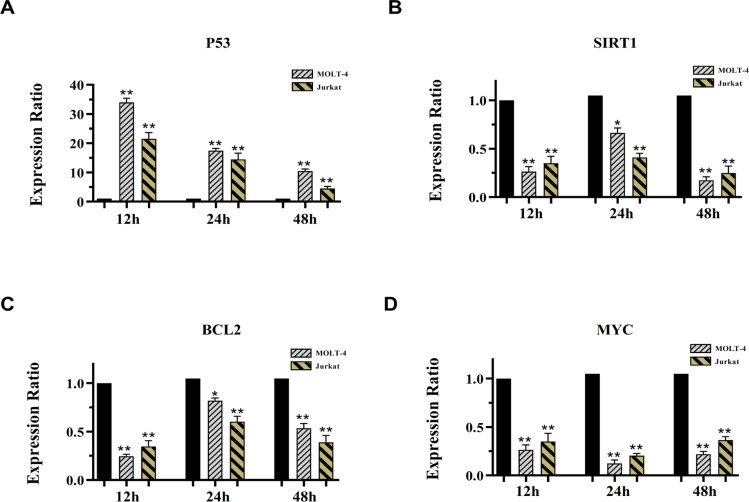
Real Time-PCR data for P53 (A), SIRT1 (B), BCL2 (C) and MYC (D) Expression. Expression levels of the candidate genes were evaluated in MOLT4 cell after exposure with 16.51µM 5-AZA and in Jurkat cell after treatment with 12.81µM 5-AZA at 12, 24 and 48 hours of treatment. GAPDH was selected as internal control. Student’s t-test was used for a comparison between each pairing group. All numerical data are presented as means ± SEM of three independent experiments. P values < 0.05 were considered to be statistically significant. (* ; p < 0.05, ** ; p < 0.01), 5-AZA; 5-azacytidine; SEM, standard error of the mean

Given the observation of a marked shift in miRNA expression, we aimed to evaluate the putative changes elicited by 5-AZA on the expression of their target genes and the closely related effectors to establish whether alteration of the target genes corresponds to that of respective miRNAs in 5-AZA-treated ALL cancer cells. To this end, expression of several genes associated to apoptosis such as p53, SIRT1, BCL2 and MYC have been tested (Figure 4A-D). The miR-34 family is known to form a system of interconnected network with these factors operating in growth arrest, and apoptosis (Yamakuchi and Lowenstein, 2009). Our data support the idea that the modulating effect of 5-AZA involves upregulation of p53 expression, thus providing a positive mechanism for increasing miR-34 transcription in MOLT4 and Jurkat cell line. The results showed that prolonged 5-AZA treatment led to a reduction of p53 mRNA in target cells (Figure 4A). Numerous studies have implicated a role for SIRT1 in suppressing the p53 function to block apoptotic progression (Yi and Luo, 2010b) whose expression has been shown to be elevated during leukemogenesis (Chen et al., 2015; Kozako et al., 2012). Given the potential contribution of SIRT1 as a regulator of p53, further investigation of this gene was of particular interest. We found that at 12 and 48 h of cells treatment SIRT1 and BCL2 expression was considerably inhibited upon 5-AZA treatment, although it was evident that there was a trend toward higher SIRT1 and BCL2 at the 24 h (Figure 4B, C). So, we reasoned that decreased expression of SIRT1 observed here might rescue p53 from SIRT1-mediated inactivation. Furthermore, the expression level of MYC was also inhibited by 5-AZA and reached a minimum value, which represented an 4-fold decrease in respect to untreated cells over the whole time range (Figure 4D). 

*Treatment with 5-AZA impairs DNA damage response through downregulation of a DNA repair enzyme in treated cells*

**Figure 5:**
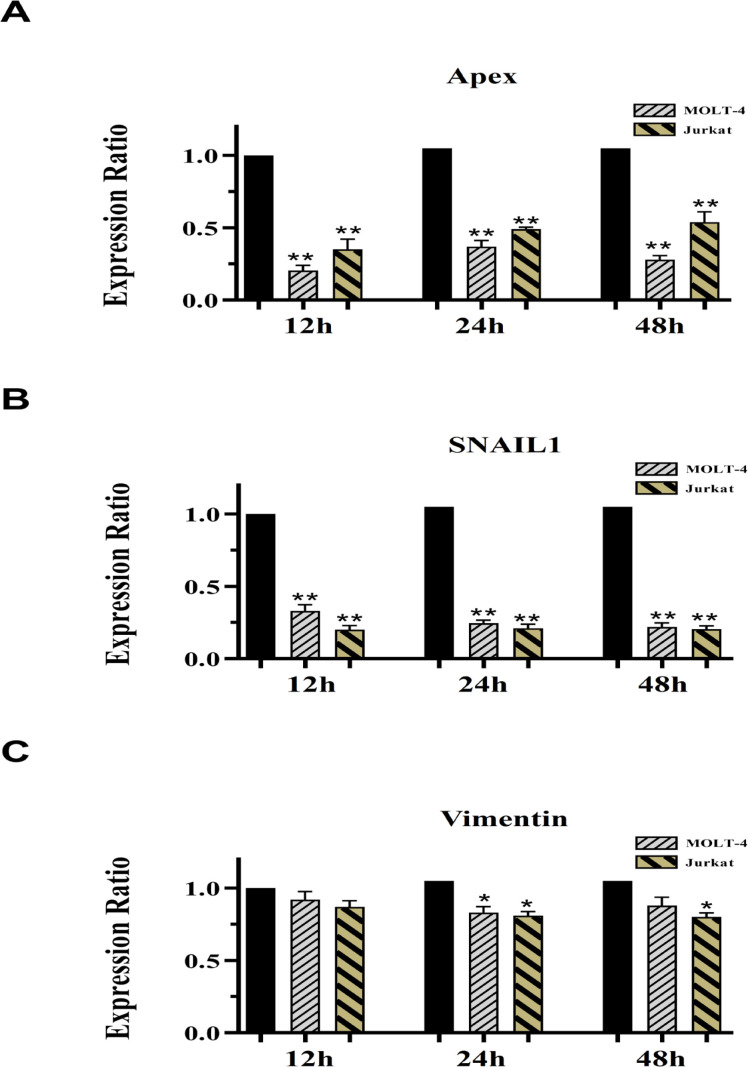
Real Time-PCR Data for APEX (A), SNAIL1 (B) and Vimentin (C) Expression. Expression levels of the candidate genes were evaluated in MOLT4 cell after exposure with 16.51µM 5-AZA and in Jurkat cell after treatment with 12.81µM 5-AZA at 12, 24 and 48 hours of treatment. GAPDH was selected as internal control. Student’s t-test was used for a comparison between each pairing group. All numerical data are presented as means ± SEM of three independent experiments. P values < 0.05 were considered to be statistically significant. (* ; p < 0.05, ** ; p < 0.01), 5-AZA; 5-azacytidine; SEM, standard error of the mean

In addition, DNA repair pathway has been indicated as a crucial process impeding the cellular responses to several cytotoxic agents. This process may represent a further mechanism by which 5-AZA might exert growth arrest. The multifunctional DNA repair enzyme Apurinic/apyrimidinic (AP) endonuclease 1 (APE1/ APEX) is responsible for the repair of DNA damage conferred by many anticancer agents. In addition to the repair of DNA damage, APEX is also associated with many transcription factors (HIF1-α, p53, AP1 and others) (Tell et al., 2005). Many studies indicate that APEX expression is constitutively activated in cancer cells suggesting that tumors having higher APEX expression preserve genome integrity and may provide protection against genotoxic impacts of endogenous and exogenous compounds (Raffoul et al., 2012). Therefore, downregulation of the DNA repair activity by inhibition of APEX is required for cell death and can enhance cancer therapy and prevention. The results of this investigation provide indirect evidence for a primary effect of 5-AZA on APEX expression as seen by decreased mRNA expression of APEX in MOLT4 and Jurkat cells in vitro following treatment with 5-AZA (Figure 5A). Based analysis, this reduction in 12 h of treatment was prominent than 24 and 48 h of treatments (Figure 5A).

*5-AZA may act to influence tumor cell invasion by regulating SNAIL1 and Vimentin expression in treated cells*

We sought to determine if the treatment had any effect on cell migration and invasion which is a late event in tumor progression. In the next experiment, we examined the time-dependent effect of 5-AZA on the mRNA expression of SNAIL1 and vimentin in MOLT4 and Jurkat cell line. The transcription factor SNAIL1 is an inducer of epithelial-to-mesenchymal transition (EMT) program and vimentin is a marker prerequisite for cell motility resulting in malignant cells (De Herreros et al., 2010; Satelli and Li, 2011), both of which may be modulated by the addition of 5-AZA. Consistent with this possibility, it was observed that the level of SNAIL1 was significantly reduced within 12 h and remained low over the next 48 h in MOLT4 and Jurkat cells (Figure 5B). By contrast, total vimentin mRNA was relatively unaffected with a slight decrease being detected at 24 h of incubation of MOLT-4 cells (Figure 5C). On the other hand, 5-AZA decreased vimentin expression levels within 12 and 48, but not 24 h of treatment of Jurkat cells (Figure 5C).

## Discussion

Epigenetic modifications, such as DNA hypermethylation have been described as a key event in the development of ALL that result in transcriptional gene suppression. Additionally, DNA methylation status is one contributory factor in the treatment of ALL cancer. The altered DNA methylation as a hallmark of varied types of cancer types is supposed to be associated with the deregulation of DNA methyltransferases. Researches have shown that 5-AZA has the potential to restore the expression of epigenetically-silenced tumor suppressor genes (Christman, 2002). Tumor-specific downregulation of several microRNA (miRNA) species has been detected in virtually every type of human neoplasia and contributes to the establishment of these malignancies. Aberrant promoter methylation of tumor suppressor miRNA genes has been described as an early driver event in ALL leukemogenesis. miRNAs are small regulatory molecules involved in several processes, such as proliferation, cell cycle regulation and apoptosis.

Based on reports, 5-AZA successfully sustained the second remission in patients suffered with ALL who relapsed following unrelated cord blood transplantation (Chijimatsu et al., 2017). Also, Nicholas et al. found that long-term treatment of leukemia cells with dasatinib and 5-AZA strongly attenuated proliferation of these cells through upregulation of miR-217 and suppression of DNMT3A expression in vitro (Nishioka et al., 2014). It seems that there exist correlation between target miRs and p53 expression, because it has been found that miRNAs like miR-34a and miR-34b can eventually contribute to the p53-mediated activities (He et al., 2007). In this regard, the miR-34 family, through SIRT1 pathway, amplifies p53 response indicating a positive feedback loop between miR-34 and p53 signaling. SIRT1 is a NAD+-dependent deacetylase inhibitor of p53 and has been likewise identified as a direct target of miR-34. Deacetylation of p53 by SIRT1 prevents apoptosis induction, and thereby support the shift towards cell survival (Yang, Fu, Pestell, and Sauve, 2006). The level of SIRT1 is also markedly elevated in cancer cells such as acute and chronic leukemic cells, prostate and breast carcinoma (Huffman et al., 2007; Lee et al., 2011; Li and Bhatia, 2015). In this study, we have described that DNA methylation-based therapy using 5-AZA was able to set the correct early expression of the majority of genes at the mRNA level in vitro. In line with these data, miRNA profile of MOLT4 cells showed substantial changes for miR-34a, miR-34b and miR-124-1 in expression favoring cell cycle arrest during 5-AZA treatment. These findings may provide additional information for better understanding of epigenetic transcriptional regulation of the multiple genes acting at different aspects of growth control in acute lymphoblastic leukemia cells. We observed a significant cytotoxicity in MOLT4 and Jurkat cells when treated with 5-AZA which was paralleled by an increased p53 expression, a decreased SIRT1, inhibition of MYC transcription and downregulation of BCL2 mRNA expression (Fig.3). 5-AZA, having hypomethylating activity, also produced high yields of the selected miRNAs and the analyzed miRNA products proved to have a negative correlation with their corresponding target genes. Overall, as statistical analysis have showed a significant correlation between *p53* and *BCL2* expression in leukemia cells (Sahu and Jena, 2011), we guess that up regulation of p53 expression after exposure with 5-AZA in turn led to the BCL2 down regulation, which finally triggered apoptosis in MOLT4 and Jurkat cells. Fourthermore, it has been suggested that p53 play a central role in SIRT1-mediated functions in tumorigenesis and there exist association between these expression and function (Yi and Luo, 2010a). Here we provide evidence that 5-AZA treatment enhances susceptibility to apoptotic cell death, and that these effects are associated with decreased SIRT1 expression in MOLT4 and Jurkat cell line. These changes were accompanied by dramatic increase in p53 and the intracellular miR-34 content, further suggesting that targeting the miR34/ SIRT1/ p53 signaling pathway is achieved by the use of 5-AZA. Consistently, a recent study by Cao et al. found that miR34b expression is downregulated in several acute leukemia cell lines as well as primary leukemic cells from ALL patients and detected CpG island methylation as a mechanism responsible for *miR-34b* gene silencing. Further, they confirmed that restoration of miR-34b expression in K562 cells by the transfection of miR34b mimics suppressed their proliferation (Cao et al., 2016). By integrating miRNA gene expression analysis and mRNA profiling, we characterized the role of miR-34 encoding genes in the T-cell leukemia cell line, MOLT4 cells. Multiple methods have been used by researchers in order to induce the expression of miR-34 including synthesized miR-34a mimics (Wiggins et al., 2010). But yet, the search for miR-34 therapeutics continues. Due to the epigenetic repression of miR-34 family within CpG islands in cancer, treatment with the methyltransferase inhibitor 5-AZA is expected to overexpress the miR-34. On the other hand, the signaling pathway commencing with miR-34 reexpression may also engage p53-independent mechanisms as evidenced by *MYC* and *BCL2* gene suppression (Figure 3). It has been reported that deregulated MYC promotes cellular proliferation and BCL2 acts to block cell death. In addition, others have shown that MYC mRNA has a predicted target site for miR-34 in its 3′ UTR (Kong et al., 2008) demonstrating that the oncogene MYC is a direct target of miR-34 (Wei et al., 2008). Specifically, MYC plays an important regulatory role in the modulation of p53-dependent apoptosis (Junttila and Evan, 2009). The data obtained here showed that in MOLT4 and Jurkat cells treated with 5-AZA the expression of MYC mRNA was strongly down-regulated. However, it was not clear whether decline in MYC expression actually do enhance cytotoxic effect of 5-AZA because MYC is also found to be a potent inducer of apoptosis (Heiko Hermeking and Eick, 1994). Furthermore, another direct target of miR-34 is BCL2, which has been correlated with lower survival (Campana et al., 1993) and high risk features (Kaparou et al., 2013) in acute lymphoblastic leukemia. The present results indeed show a modest but significant decrease in the levels of BCL2 mRNA relative to those in controls. ALL leukemia cells generally depend on BCL2 for their survival (Moore, Schlis, Sallan, Armstrong, and Letai, 2008), therefore, a slight diminish in BCL2 expression levels, might significantly affect cell survival in these cells.

Moreover, 5-AZA -induced cytotoxicity can also result from DNA damage (Palii, Van Emburgh, Sankpal, Brown, and Robertson, 2008). It has been previously established that 5-AZA treatment causes DNA double-strand breaks and this effect proved to be reversible (Palii et al., 2008). Other studies addressing master regulators controlling various signaling pathways in leukemia found that the multifunctional protein redox factor-1 (Ref1/APE1) may serve as a signaling node through its regulation of transcription factors important in T-ALL (Ding et al., 2017). Analysis of the transcriptomes of patients’ leukaemic cells revealed increased expression of *APEX* and other genes of the Ref1 pathway in T-ALL (Ding et al., 2017). Reduction of APEX nuclease activity and/or its expression is of functional importance since the Ref-1 is involved in growth control and the DNA damage response of tumor cells. The APEX was originally characterized as an endonuclease which cleaves the backbone of double-stranded DNA breaks to continue the DNA repair process. If the damage cannot be repaired, these lesions can lead to cell death promotion by blocking DNA replication (Schaaper, Kunkel, and Loeb, 1983). Intriguingly, this protein is a substrate of SIRT1 deacetylase. It is now evident that SIRT1 physically interacts and deacetylates the APEX protein and thereby facilitates cytoprotection against genotoxic stress. This suggests that SIRT1 cooperates with APE1 to protect from cell death induced by genotoxic stimuli (Yamamori et al., 2009). Based on our experimental findings reversal of DNA methylation by 5-AZA could notably suppress the levels of APEX transcripts and as APEX levels decreased, so did levels of SIRT1. Thus, it was likely that the cytotoxic functions of 5-AZA may be possibly related to an modified expression of SIRT1 mRNAs in MOLT4 cells. Finally, some of the predicted *miR-34* target genes encode for proteins that have important role in cell motility and invasion such as *SNAIL1* genes. SNAIL1 family members function as transcriptional repressors that trigger acquisition of invasive and migratory properties during EMT (Nieto, 2002). According our results we approve the previous findings that repressing miR-34a and miR-34b/c led to upregulation of SNAIL1 expression (Siemens et al., 2011). In this work, SNAIL1 mRNA showed a drastic decrease after 5-AZA treatment that coincides with a change in the expression pattern of miR-34. Of course, existence of the decisive correlation between miR-34 expression and EMT progression needs to be studied further in leukemia cells. Moreover, this study highlights the potential of DNA demethylating treatment to reactivate hypermethylated miR-124-1 in MOLT4 and Jurkat cells. This is certainly consistent with data from Lujambio et al., (2007) who first reported the epigenetic silencing of miR-124 in ALL. In another study, Wong et al., (2011) demonstrated that demethylating effect of 5-AZA could result in re-expression of mature miR-124 in myeloma cells. Based results, we found that there is significant, but not powerful association between target miRs and vimentin expression, which could modify leukemic cells growth and metastasis.

It is therefore tempting to suggest that mechanisms underlying the 5-AZA effects not only control the induction of apoptotic features, but may also reduce the risk of metastasis of ALL cancer cells through downregulation of SNAIL1 and vimentin transcripts. Indeed, our preliminary observations suggest that miR-34 augmentation with 5-AZA treatment may induce different apoptotic pathways via a three-step pathway; a p53 stimulated process, a transcription inhibition of APEX and a BCL2-mediated mechanism. Notably, our results clearly indicate that the therapeutic activity observed in 5-AZA -treated MOLT4 and Jurkat cells require functional p53. This suggests that 5-AZA therapy could be of therapeutic use for ALL patients, since the majority of ALL tumors typically retain wild-type p53.

## Author Contribution Statement

The authors confirm contribution to the paper as follows: study conception and design: Saeed Solali and Mohammad Reza Alivand; data collection: Behnam Emamgolizadeh and Dara RahmanPour; analysis and Interpretation of results: Ali Mohammadi and behnam Emamgolizadeh; draft manuscript preparation: Behnam Emamgolizadeh and Ali mohammadi. Dara Rahmanpour revised the article. All authors reviewed the results and approved the final version of the manuscript.

## Ethical Approval 

The ethical committee of Tabriz University of Medical Science has approved this study. 

## Funding

This study was funded by Tabriz University of Medical Sciences.

## Data availability statement

All data for this study are available.

## Conflict of Interests

The authors declare no conflict of interests.
